# Impaired Hippocampal Glutamate and Glutamine Metabolism in the db/db Mouse Model of Type 2 Diabetes Mellitus

**DOI:** 10.1155/2017/2107084

**Published:** 2017-06-14

**Authors:** Jens Velde Andersen, Jakob Dahl Nissen, Sofie Kjellerup Christensen, Kia Hjulmand Markussen, Helle Sønderby Waagepetersen

**Affiliations:** Department of Drug Design and Pharmacology, Faculty of Health and Medical Sciences, University of Copenhagen, Copenhagen, Denmark

## Abstract

Type 2 diabetes mellitus (T2DM) is a risk factor for the development of Alzheimer's disease, and changes in brain energy metabolism have been suggested as a causative mechanism. The aim of this study was to investigate the cerebral metabolism of the important amino acids glutamate and glutamine in the db/db mouse model of T2DM. Glutamate and glutamine are both substrates for mitochondrial oxidation, and oxygen consumption was assessed in isolated brain mitochondria by Seahorse XFe96 analysis. In addition, acutely isolated cerebral cortical and hippocampal slices were incubated with [U-^13^C]glutamate and [U-^13^C]glutamine, and tissue extracts were analyzed by gas chromatography-mass spectrometry. The oxygen consumption rate using glutamate and glutamine as substrates was not different in isolated cerebral mitochondria of db/db mice compared to controls. Hippocampal slices of db/db mice exhibited significantly reduced ^13^C labeling in glutamate, glutamine, GABA, citrate, and aspartate from metabolism of [U-^13^C]glutamate. Additionally, reduced ^13^C labeling were observed in GABA, citrate, and aspartate from [U-^13^C]glutamine metabolism in hippocampal slices of db/db mice when compared to controls. None of these changes were observed in cerebral cortical slices. The results suggest specific hippocampal impairments in glutamate and glutamine metabolism, without affecting mitochondrial oxidation of these substrates, in the db/db mouse.

## 1. Introduction

Type 2 diabetes mellitus (T2DM) is a multifaceted metabolic disease characterized by an augmented level of blood glucose caused by insulin resistance. T2DM has reached pandemic proportions as over 400 million people are affected by diabetes [1]. T2DM is a major risk factor for the development of Alzheimer's disease (AD), and the hippocampus seems to be particularly vulnerable in T2DM [2–4]. Insulin resistance has been associated with reductions in cerebral glucose utilization, a clinical hallmark of AD [5, 6]. This observation suggests that altered brain energy metabolism might be an accelerating factor in the development of AD in T2DM.

Glutamate is the main excitatory neurotransmitter in the mammalian brain. Timely clearance and handling of glutamate is essential for normal cerebral function [7]. The majority of released neurotransmitter glutamate is removed from the synapse by adjacent astrocytes. In the astrocyte, glutamate can be converted into glutamine by the astrocyte-specific enzyme glutamine synthetase (GS). Glutamine can subsequently be transported to the neurons and converted back into glutamate by the enzyme phosphate-activated glutaminase (PAG), mainly expressed in the neurons. The exchange of glutamate and glutamine between neurons and astrocytes is termed the glutamate/glutamine cycle [7, 8]. Glutamate and glutamine are also linked to cellular energy homeostasis. Glutamate can be converted into *α*-ketoglutarate by activity of glutamate dehydrogenase (GDH) or aspartate aminotransferase (AAT) and thus be oxidized in the tricarboxylic acid (TCA) cycle [9–11]. Glutamine must be converted into glutamate prior to oxidation. Both neurons and astrocytes are able to metabolize glutamate and glutamine [12–14]. It has recently been shown that oxidation of glutamate through GDH is important for cerebral energy homeostasis [15].

Previously, we have shown that the common T2DM mouse model, the db/db mouse, exhibits reduced glucose utilization in both the cerebral cortex and hippocampus [16]. In addition to this, isolated cerebral mitochondria consumed more oxygen when given pyruvate and malate as substrates. Here, we sought to investigate if cerebral glutamate and glutamine metabolism is altered in the db/db mouse. To assess this, oxygen consumption, that is, respiration, of isolated whole-brain mitochondria was determined by Seahorse XFe96 analysis, providing glutamate and glutamine as primary substrates. Furthermore, acutely isolated cerebral cortical and hippocampal slices of control and db/db mice were incubated in media containing ^13^C-labeled glutamate or glutamine and tissue extracts were subsequently analyzed by gas chromatography-mass spectrometry (GC-MS) to determine ^13^C enrichment and map the metabolism. Finally, the expression of enzymes important for glutamate/glutamine homeostasis was assessed by Western blotting.

## 2. Methods

### 2.1. Materials

The stable isotopes [U-^13^C]glutamate and [U-^13^C]glutamine (both L isoform and 99% purity) were purchased from Cambridge Isotope Laboratories (Tewksbury, MA, USA). The following antibodies were purchased from Abcam (Cambridge, United Kingdom): glutamate dehydrogenase (GDH, rabbit: ab34786), aspartate aminotransferase (AAT, mouse: ab93928), glutamate decarboxylase 65 and 67 (GAD65 and 67, both mouse: ab26113 and ab26116), phosphate-activated glutaminase (PAG, rabbit: ab93434), and glutamine synthetase (GS, rabbit: ab73593). All other chemicals used were of the purest grade available from regular commercial sources.

### 2.2. Animals

Diabetic and obese homozygote db/db and control mice (blood glucose: 25.6 ± 1.3 mmol/L versus 11.1 ± 0.4 mmol/L, body weight: 51.6 ± 0.8 g versus 28.2 ± 0.9 g) were obtained through mating of heterozygote (db/−) mice at the Department of Drug Design and Pharmacology, University of Copenhagen. The breeding pairs were obtained from Jackson Laboratories (Bar Harbor, ME, USA) on a C57BL/6J background. All animals were kept in a humidity-controlled facility with 12/12 h light/dark cycle and free access to water and chow. Animals were used at 15-16 weeks of age and grouped according to obese phenotype, meaning both lean wild-type and heterozygote offspring were used as controls as they cannot be phenotypically distinguished [17, 18]. Both male and female mice were included in this study. Blood glucose levels were assessed using a BAYER Breeze 2 instrument. The experiments were approved by the Danish National Ethics Committee and were performed in agreement with the European Convention (ETS 123 of 1986).

### 2.3. Mitochondrial Isolation and Seahorse XFe96 Analysis

Whole-brain mitochondria of control and db/db mice were isolated in tandem as described previously [16]. Briefly, animals were euthanized by cervical dislocation, the brain quickly removed and placed in cold isolation buffer containing the following in mM: mannitol 210, sucrose 70, HEPES 5, EGTA 1 and 0.5% BSA (fatty acid free), pH 7.2, and homogenized using a Teflon douncer (750 revolutions/min, 7-8 strokes). The mitochondria in the homogenate were isolated using a 21% Percoll gradient, and protein amounts were determined by the Bradford method. The oxygen consumption rate (OCR) of the isolated cerebral mitochondria was assessed at 37°C using a Seahorse XFe96 analyzer (Seahorse Biosciences, MA, USA). The isolated mitochondria were suspended in assay buffer containing the following in mM: mannitol 220, sucrose 70, KH_2_PO_4_ 10, MgCl_2_ 5, HEPES 2, and 0.2% BSA (fatty acid free), pH = 7.2. An aliquot of 25 *μ*L mitochondrial suspension, containing 4 *μ*g of protein, was seated in each well and the plate was centrifuged (2.000*g* × 20 min at 4°C). The mitochondria were provided with 37°C assay buffer containing 10 mM glutamate or 10 mM glutamine both in the presence of 10 mM malate (all final concentrations) and analyzed immediately. Malate was provided to support the pool of TCA cycle intermediates. The sequence of analysis consisted firstly of a waiting period of 10 min, followed by two cycles of mixing (1 min) and waiting (3 min). Subsequently, three cycles of mixing (2 min), waiting (1 min), and measurements (3 min) were applied to establish the baseline OCR. During the subsequent course of measurements, four compounds were injected, in the following order: ADP (4 mM), oligomycin A (2.5 *μ*g/mL), carbonyl cyanide-p-trifluoromethoxyphenylhydrazone (FCCP, 8 *μ*M), and antimycin A (8 *μ*M)—all final concentrations. OCRs were calculated using the Wave software (Seahorse Biosciences). Relative OCR levels were calculated by setting the OCR at the third baseline measurement to 100%.

### 2.4. Preparation and Incubation of Acute Brain Slices

Acutely isolated brain slices were prepared as previously described [19, 20]. Briefly, animals were euthanized by cervical dislocation and the brain excised into ice-cold artificial cerebrospinal fluid (ACSF) containing the following in mM: NaCl 128, NaHCO_3_ 25, KCl 3, CaCl_2_ 2, MgSO_4_ 1.2, and KH_2_PO_4_ 0.4, pH = 7.4. The cerebral cortices and hippocampi were sliced using a McIlwain Tissue Chopper (The Vibratome Company, O'Fallon, MO, USA) into 350 *μ*m thick slices. The slices were incubated in 10 mL ACSF containing 10 mM D-glucose for 60 min to recover from the slicing. Subsequently, the media were exchanged for ACSF containing 0.5 mM [U-^13^C]glutamate or 1 mM [U-^13^C]glutamine both in combination with 5 mM D-glucose, and the slices were incubated for 60 min. The incubations were terminated by transferring slices to ice-cold 70% ethanol. Slice were sonicated and centrifuged (20.000*g* × 20 min) and the supernatant was lyophilized and stored for later analysis. The pellets were saved for protein determination by the Pierce method. The dry brain slice extracts were reconstituted in H_2_O for determination of ^13^C enrichment and amino acid amounts by GC-MS and HPLC analysis, respectively.

### 2.5. Metabolic Mapping by Gas Chromatography-Mass Spectrometry (GC-MS) Analysis

**Figure 1:**
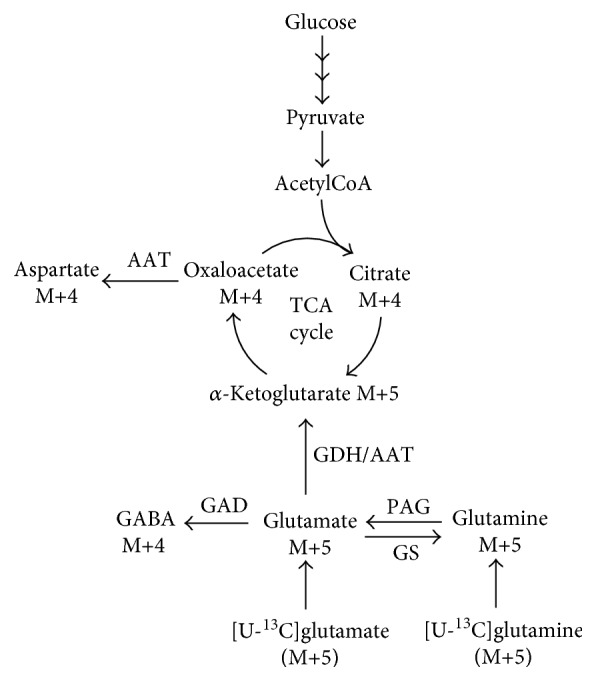
Cartoon illustrating ^13^C labeling patterns from [U-^13^C]glutamate and [U-^13^C]glutamine metabolism. Glutamate M+5 can be converted into glutamine M+5 or GABA M+4 through glutamine synthetase (GS) or glutamate decarboxylase (GAD) activity, respectively. Glutamine M+5 can likewise be converted into glutamate M+5 through the action of phosphate-activated glutaminase (PAG). Additionally, glutamate M+5 can be converted into *α*-ketoglutarate M+5 by activity of either glutamate dehydrogenase (GDH) or aspartate aminotransferase (AAT) and be introduced into the TCA cycle. *α*-Ketoglutarate M+5 can be metabolized in the TCA cycle, giving rise to ^13^C labeling in all TCA cycle intermediates. Aspartate is in close equilibrium with oxaloacetate trough AAT, giving rise to aspartate M+4 labeling.

GC-MS analysis of ^13^C enrichment in amino acids and metabolites from brain slice extracts was performed as described by Walls et al. [21]. The natural abundance of ^13^C was taken into account using data obtained from analysis of standards containing unlabeled metabolites of interest. Data are presented as the % ^13^C enrichment of *M* + *X*, where *M* is the molecular ion of the given metabolite and *X* is the number of ^13^C carbon atoms in the molecule. The expected pattern of ^13^C labeling from incubations with [U-^13^C]glutamate and [U-^13^C]glutamine is given in Figure 1.

### 2.6. Amino Acid and Lactate Determination

Determination of quantitative amounts of amino acids from brain slice extracts was assessed by reversed-phase high-performance liquid chromatography (HPLC) using precolumn o-phthalaldehyde derivatization and fluorescent detection (excitation *λ* = 338 nm, emission *λ* = 390 nm) as previously described [19]. The amounts of lactate released from the brain slices to the incubation medium were assessed using an enzymatic kit, based on the coupled reactions between lactate dehydrogenase and alanine aminotransferase, from Boehringer Mannheim/R-Biopharm AG (Darmstadt, Germany) according to the manufacturer's instructions.

### 2.7. Western Blots

Cerebral cortical and hippocampal homogenates of db/db and control mice were analyzed by Western blotting using primary antibodies against GDH, AAT, glutamate decarboxylase (GAD), PAG, and GS. Equal amounts (30 *μ*g) of protein were separated by SDS-PAGE using NuPAGE 4–12% Bis-Tris gels (Thermo Fisher Scientific) before transferring the proteins to PVDF membranes. The membranes were stained with Ponceau S solution for validation of equal protein amounts, washed, and incubated in the given primary antibodies, and appropriate HRP-conjugated secondary antibodies (Dako) were used. The blots were subsequently analyzed with ECL™ Prime Western Blotting Detection Reagent and quantified using ImageJ software.

### 2.8. Data Presentation and Statistical Analysis

All data are presented as means ± standard error of the mean (SEM). To test if differences between the control and db/db groups were statistically significant, Student's *t*-test were employed. *P* values of <0.05 were considered to be significant and are indicated with a single asterisk.

## 3. Results

### 3.1. Mitochondrial Oxidation of Glutamate and Glutamine

**Figure 2:**
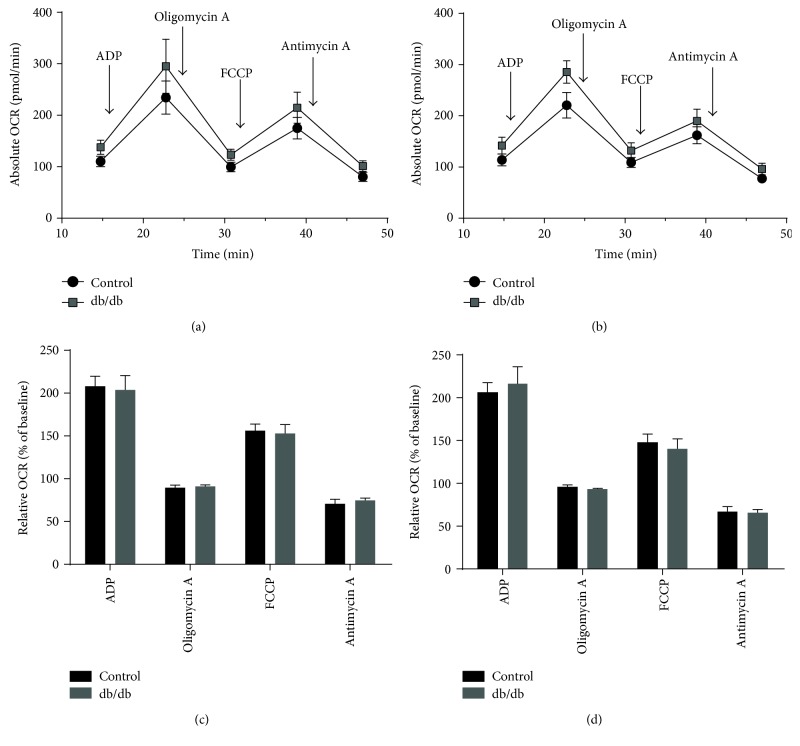
Mitochondrial oxygen consumption rates (OCRs) of isolated brain mitochondria from control and db/db mice. Absolute (a and b) and relative (c and d) oxygen consumption rates of the brain mitochondria from control and db/db mice, with glutamate (a and c) and glutamine (b and d), both in the presence of malate, as substrates. Arrows (in a and b) indicate the specific time points of compound addition. ADP stimulates coupled respiration, oligomycin A inhibits the ATP synthase, FCCP induces uncoupled respiration, and antimycin A inhibits complex III of the electron transport chain. Baseline is not shown. Results are presented as means ± SEM, *n* = 4–7, Student's *t*-test, *p* < 0.05.

To investigate if the cerebral mitochondrial oxidation of glutamate and glutamine was altered in the db/db mice, oxygen consumption rates (OCRs) of isolated whole-brain mitochondria were assessed with glutamate and glutamine as substrates, both in the presence of malate. Glutamine is taken up by the mitochondria and transformed into glutamate, which can be converted into *α*-ketoglutarate and support oxidative metabolism in the TCA cycle. Absolute and relative OCRs of isolated whole-brain mitochondria from control and db/db mice are presented in Figure 2. Addition of ADP stimulates coupled respiration, which can be blocked by the ATP synthase inhibitor oligomycin A. FCCP induces maximal uncoupled respiration, whereas antimycin A inhibits complex III of the respiratory chain halting the flow of electrons. No significant changes were observed in the OCR of mitochondria from control and db/db mice when provided with glutamate (Figure 2(a)) or glutamine (Figure 2(b)) as respiratory substrates in the presence of any of the four compounds. A tendency towards an overall higher OCR was observed in the mitochondria of db/db mice for both substrates. However, these tendencies were not present in the relative OCR levels (Figures 2(c) and 2(d)), suggesting little or no changes in cerebral mitochondrial utilization of glutamate and glutamine in the db/db mouse.

### 3.2. Amino Acid Amounts of Isolated Cerebral Cortical and Hippocampal Slices

**Figure 3:**
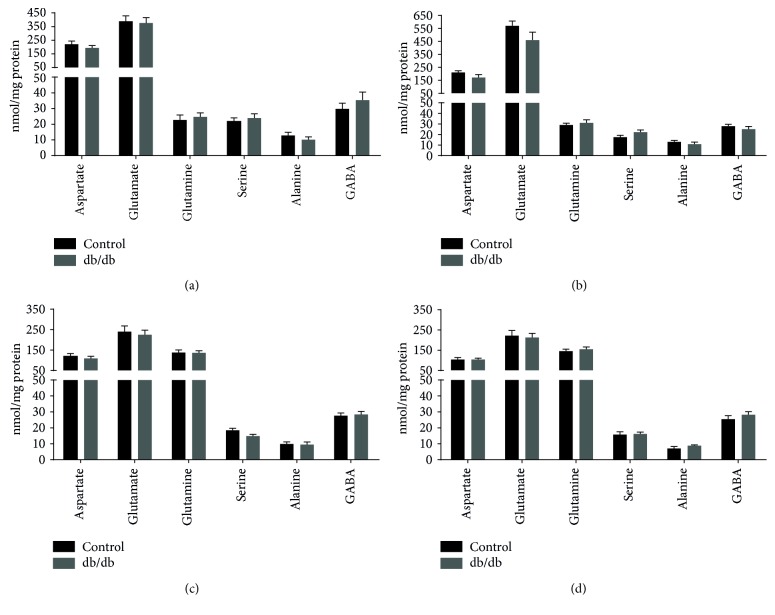
Amino acid amounts in extracts of cerebral cortical and hippocampal slices of control and db/db mice. Amino acid amounts of cerebral cortical (a and c) and hippocampal (b and d) slices of control and db/db mice incubated in media containing either [U-^13^C]glutamate (a and b) or [U-^13^C]glutamine (c and d). Results are presented as means ± SEM, *n* = 8–11, Student's *t*-test, *p* < 0.05.

The content of amino acids of cerebral cortical and hippocampal slices from control and db/db mice incubated in media containing [U-^13^C]glutamate or [U-^13^C]glutamine is presented in Figure 3. No significant changes were observed in amino acid amounts of cerebral cortical and hippocampal slices incubated in media containing [U-^13^C]glutamate (Figures 3(a) and 3(b)). A tendency towards lower glutamate levels was observed in the hippocampal slices of db/db mice (*p* = 0.12). Likewise, no significant changes in amino acid amounts were observed for cerebral cortical and hippocampal slices of control and db/db mice incubated in media containing [U-^13^C]glutamine (Figures 3(c) and 3(d)). In addition, no significant changes in lactate amounts released to the media during the incubations were observed (data not shown).

### 3.3. Metabolism of [U-^13^C]Glutamate in Acute Brain Slices

**Figure 4:**
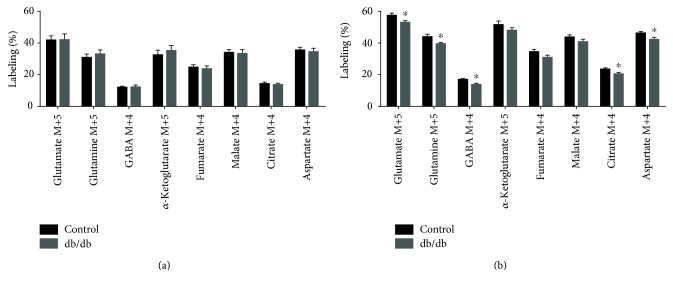
^13^C labeling from [U-^13^C]glutamate metabolism of cerebral cortical and hippocampal slices of control and db/db mice. TCA cycle intermediates and amino acids labeled from [U-^13^C]glutamate metabolism during incubations of cerebral cortical (a) and hippocampal (b) slices of control and db/db mice. Results are presented as means ± SEM, *n* = 8–11, Student's *t*-test, ^∗^*p* < 0.05.

Even though no changes in mitochondrial oxidation of glutamate and glutamine were found in the db/db mouse, more subtle regional changes could be present. To detect such changes, acutely isolated cerebral cortical and hippocampal slices were incubated in media containing [U-^13^C]glutamate or [U-^13^C]glutamine, and isotopic enrichment was assessed in tissue extracts by GC-MS analysis. The expected labeling pattern of [U-^13^C]glutamate metabolism is depicted in Figure 1. The ^13^C enrichment of amino acids and TCA cycle intermediates from [U-^13^C]glutamate metabolism in cerebral cortical and hippocampal slices from control and db/db mice is presented in Figure 4. No apparent changes were observed in ^13^C labeling from [U-^13^C]glutamate metabolism of cerebral cortical slices from control and db/db mice (Figure 4(a)). In contrast, ^13^C enrichment of several metabolites was lower in hippocampal slices of db/db mice when compared to controls (Figure 4(b)). Labeling of glutamate M+5 was significantly reduced (8%, *p* = 0.044). Additionally, enrichment in glutamine M+5 and GABA M+4 was significantly decreased (10%, *p* = 0.048 and 19%, *p* = 0.0087, resp.) in hippocampal slices of db/db mice compared to controls. Since glutamate M+5 is the direct precursor of glutamine M+5 and GABA M+4, the reduced labeling of the latter two is likely a reflection of the decreased glutamate M+5 labeling. Furthermore, significant decreases in citrate M+4 (13%, *p* = 0.045) and aspartate M+4 (9%, *p* = 0.037) were observed from [U-^13^C]glutamate metabolism in the hippocampus of db/db mice. These results suggest an impaired metabolism of glutamate, which could be linked to reduced uptake of glutamate in hippocampal slices of db/db mice.

### 3.4. Metabolism of [U-^13^C]Glutamine in Acute Brain Slices

**Figure 5:**
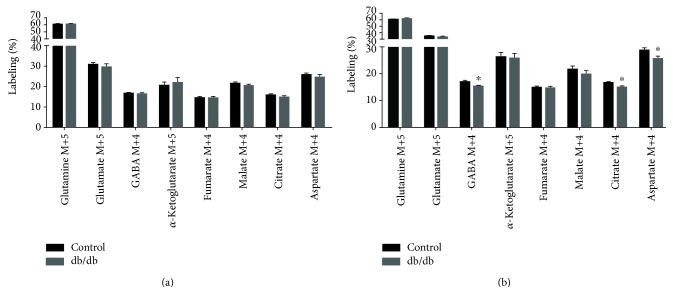
^13^C labeling from [U-^13^C]glutamine metabolism of cerebral cortical and hippocampal slices of control and db/db mice. TCA cycle intermediates and amino acids labeled from [U-^13^C]glutamine metabolism during incubations of cerebral cortical (a) and hippocampal (b) slices of control and db/db mice. Results are presented as means ± SEM, *n* = 11, Student's *t*-test, ^∗^*p* < 0.05.

The anticipated labeling pattern from metabolism of [U-^13^C]glutamine is illustrated in Figure 1. ^13^C labeling of amino acids and TCA cycle intermediates from [U-^13^C]glutamine metabolism in cerebral cortical and hippocampal slices from control and db/db mice are presented in Figure 5. No significant differences in ^13^C labeling were observed in extracts of cerebral cortical slices of control and db/db mice. However, as seen for the incubations with [U-^13^C]glutamate, several differences were found in extracts of hippocampal slices of db/db mice incubated with [U-^13^C]glutamine. No significant changes were observed in glutamine M+5 or glutamate M+5, suggesting unaltered glutamine uptake and PAG activity. However, labeling of GABA M+4 was significantly decreased (10%, *p* = 0.045) in db/db mice compared to controls. Furthermore, labeling of citrate M+4 and aspartate M+4 was significantly reduced (10%, *p* = 0.027 and 11%, *p* = 0.034, resp.) in hippocampal slices of db/db mice. These results point towards hampered metabolism of glutamine, downstream of glutamate, in the hippocampus of db/db mice.

### 3.5. Expression Levels of Enzymes in the Metabolism of Glutamate and Glutamine

The observed changes in ^13^C labeling from metabolism of [U-^13^C]glutamate and [U-^13^C]glutamine could be due to altered expression of enzymes involved in glutamate and glutamine turnover illustrated in Figure 1. However, no changes were observed in the expressions of GDH, AAT, GAD, PAG, or GS in cerebral cortical and hippocampal homogenate of db/db and control mice (see Supplementary Figure 1 available online at https://doi.org/10.1155/2017/2107084).

## 4. Discussion

In this study, we show that the mitochondrial capacity for oxidative metabolism of glutamate and glutamine in the db/db brain is maintained. However, hippocampal slices of db/db mice exhibited hampered metabolism of glutamate and glutamine, observed as decreased ^13^C labeling of several key amino acids and TCA cycle intermediates. These results suggest that both glutamate uptake and glutamine metabolism are disturbed in the hippocampus of db/db mice, potentially affecting the homeostasis of the glutamate/glutamine cycle.

### 4.1. Cerebral Mitochondrial Function in Type 2 Diabetes

In the present study, we show no significant changes in oxygen consumption of brain mitochondria from db/db mice when provided with glutamate and glutamine, suggesting little or no change in cerebral mitochondrial oxidation of these substrates. Other studies have found impaired cerebral mitochondrial function in the brain of the db/db mouse, mainly manifested as decreased expression or activity of the individual electron transport chain complexes in both the cerebral cortex and hippocampus [22, 23]. Huang et al. have recently shown an age-related decline of complex I activity in the hippocampus of db/db mice [23]. Other studies have, likewise, found reduced hippocampal gene and protein expression related to mitochondrial energetics in db/db mice [24, 25], all suggesting that the mitochondria of the hippocampus are affected by T2DM. However, oxygen consumption, being a measure of TCA cycle metabolism, electron transport, and oxidative phosphorylation, does not suggest any functional alterations in oxidative metabolism of glutamate and glutamine in the db/db brain. We have recently shown, using the same experimental setup, an augmented ADP-stimulated respiration from isolated whole-brain mitochondria of db/db mice when given pyruvate and malate as substrates [16]. This could suggest mitochondrial compensatory mechanisms at the level of oxidative phosphorylation in the db/db brain. The enhanced mitochondrial function might try to counterbalance the observed cerebral hypometabolism of glucose in db/db mice [16]. Our previous observations are in line with the results presented in this study, showing that mitochondrial oxidative function in the db/db brain is maintained. However, it should be noted that the mitochondrial preparation used in this study, as done previously, was obtained from whole-brain homogenate, meaning that potential regional differences in mitochondrial function might be masked.

### 4.2. Glutamate/Glutamine Cycling in the Diabetic Brain

Homeostasis of the glutamate/glutamine cycle is important for normal brain function and has been shown to be altered in T2DM animal models [26, 27]. Impairments of glutamate or glutamine uptake could be reflected as quantitative changes of these amino acids from extracts of the incubated brain slices. We have previously found that the amounts of the amino acids: aspartate, glutamate, glutamine, serine, alanine, and GABA, were unaltered in extracts of both cerebral cortical and hippocampal slices of db/db mice incubated in media containing glucose as substrate [16]. This observation is in accordance with previous findings by Makar et al. [28]. However, a recent study found significant increased levels of glutamate and decreased levels of glutamine in the hippocampus of 17-week-old db/db mice assessed by ^1^H-NMR [26]. This study also reported qualitative changes in GS and PAG expression using immunohistochemistry, coinciding with the altered levels of glutamate and glutamine [26]. Our results obtained from acutely isolated brain slices do not support such changes in enzyme expression. From the metabolism of [U-^13^C]glutamate, the enrichment of glutamate M+5 and glutamine M+5 was decreased to similar extents (8% and 10%, resp.) in hippocampal slices of db/db mice, suggesting intact GS activity. Likewise, from the metabolism of [U-^13^C]glutamine, labeling of glutamate M+5 synthesized by activity of PAG from glutamine M+5 was unaltered like its precursor in hippocampal slices of db/db mice. These observations are additionally supported by Western blot analyses, finding no alterations in GS or PAG expression in the cerebral cortex and hippocampus of db/db mice.

In the present study, we find that GABA M+4 labeling is decreased from both [U-^13^C]glutamate and [U-^13^C]glutamine metabolism in hippocampal slices of db/db mice. It has recently been suggested that the db/db mouse exhibits a reduced hippocampal expression of GAD [26]. Astrocyte-derived glutamine is the main precursor for neuronal GABA synthesis, and the significance of this has been established in our current incubation setup [29]. The decreased enrichment in GABA M+4 could be explained by an impaired transfer of astrocytic glutamine to neurons. However, since GABA M+4 enrichment was reduced from the metabolism of both [U-^13^C]glutamate and [U-^13^C]glutamine, this points towards specific alterations in GAD functionality. The expressions of both isoforms of GAD (GAD65 and GAD67) were found not to be different in hippocampal homogenate of db/db mice. This indicates decreased enzyme activity and not protein expression.

We observed a reduced enrichment of glutamate M+5 and a tendency towards lower glutamate amounts, from incubations of hippocampal slices in media containing [U-^13^C]glutamate, pointing towards decreased hippocampal glutamate uptake in db/db mice. It has been shown that the rate of astrocytic oxidation of glutamate rises with increasing exogenous glutamate concentrations [12]. In the present incubation study, 0.5 mM exogenous [U-^13^C]glutamate was applied. This concentration is relatively high, testing both glutamate uptake and metabolism for an extended period of time. Glutamate uptake is energetically expensive, as it is coupled to cotransport Na^+^, requiring high activity of the Na^+^/K^+^-ATPase to maintain ionic homeostasis [8]. Interestingly, it has been shown that db/db mice exhibits a reduced activity of the Na^+^/K^+^-ATPase in whole-brain homogenate, which could be linked to a reduced glutamate uptake [28]. Reduced uptake of glutamate has previously been described in the diabetic brain, which is in line with our results suggesting impairments in this important part of the glutamate/glutamine cycle in the hippocampus [30, 31]. Interestingly, it has been suggested that the Na^+^/K^+^-ATPase is mainly fueled by glycolytically derived ATP [32]. This might explain the decreased glutamate uptake, since we previously have shown hypometabolism of glucose in the brain of db/db mice [16].

The reduced labeling observed from incubations with [U-^13^C]glutamine could arise from reduced neuronal TCA cycle activity, as recently shown in a mouse model of early AD [19]. If the TCA cycle activity is decreased, the need for introduction of *α*-ketoglutarate derived from glutamine through glutamate likewise falls, leading to reduced ^13^C labeling. However, since only reduced labeling is observed in citrate M+4 and no other TCA cycle intermediate, this is not a likely explanation. In addition, our results indicate that glutamine uptake and conversion into glutamate are unaffected. The main enzymes converting glutamate into *α*-ketoglutarate are AAT and GDH as outlined in Figure 1 [7]. One study has reported a decreased expression of AAT in the hippocampus of db/db mice [24]. This observation is in line with our results, since aspartate M+4 labeling was reduced from the metabolism of both [U-^13^C]glutamate and [U-^13^C]glutamine, suggesting reduced activity of AAT in the hippocampus of db/db mice. The reduced labeling in citrate M+4, likewise observed for both [U-^13^C]glutamate and [U-^13^C]glutamine metabolism, is also in line with hampered AAT activity. Since aspartate is in fast equilibrium with oxaloacetate, decreased AAT activity would lead to reduced labeling of oxaloacetate M+4, which is reflected in citrate M+4. We were not able to detect any changes in either AAT or GDH expression in cerebral cortical or hippocampal homogenate of db/db mice.

### 4.3. Hippocampal Impairments in Type 2 Diabetes

We have recently shown that glucose hypometabolism is present in both the cerebral cortex and hippocampus of the db/db mouse, being more prominent in the cerebral cortex [16]. In the current study, changes in glutamate and glutamine metabolism were exclusively present in the hippocampus. It has been suggested that the hippocampus is particularly vulnerable to metabolic alterations in T2DM and this has been associated with memory deficits [4, 6]. Indeed, several studies have determined that the db/db mouse exhibits cognitive impairments [18, 33, 34]. In addition, several specific mitochondrial changes have been found in the hippocampus of db/db mice [23–25]. Alterations in electrophysiological functions, specifically decreased hippocampal long-term potentiation and depression, important for memory and learning, have been shown in the db/db mouse [23, 33, 34]. All of these specific impairments in the hippocampus of the db/db mouse are in line with our results of hippocampal changes in the glutamate/glutamine cycle, not being present in the cerebral cortex. Our results are intriguing in the context of the accelerated development of AD in relation to T2DM, and the specific hippocampal changes in glutamate and glutamine metabolism could be important factors of the underlying pathology.

## 5. Conclusions

We provide evidence that mitochondrial oxidation of glutamate and glutamine in the brain of the db/db mouse is unaltered. However, hippocampal slices of db/db mice exhibited reduced glutamate uptake and metabolism of glutamine, changes that were not observed in the cerebral cortex. Our findings suggest specific impairments in hippocampal GAD and AAT activities, likely affecting homeostasis of the glutamate/glutamine cycle, which could be linked to cognitive deficits in db/db mice.

## Supplementary Material

Supplementary material – Andersen et al. 2017 – Neural Plast.

## Abbreviations

AAT: Aspartate aminotransferase
ACSF: Artificial cerebrospinal fluid
AD: Alzheimer's disease
GAD: Glutamate decarboxylase
GDH: Glutamate dehydrogenase
GS: Glutamine synthetase
M: Molecular ion
OCR: Oxygen consumption rate
PAG: Phosphate-activated glutaminase
T2DM: Type 2 diabetes mellitus.
