# Supplementary material for: Impaired Hippocampal Glutamate and Glutamine Metabolism in the db/db Mouse Model of Type 2 Diabetes Mellitus
Source: Neural Plast. 2017 Jun 14;2017:2107084. doi: 10.1155/2017/2107084 (PMC5488168; doi:10.1155/2017/2107084)
Supplement: Supplementary file 1 — Supplementary material – Andersen et al. 2017 – Neural Plast. [file 2107084.f1.pdf]

Supplementary material – Andersen et al. 2017 – Neural Plast.

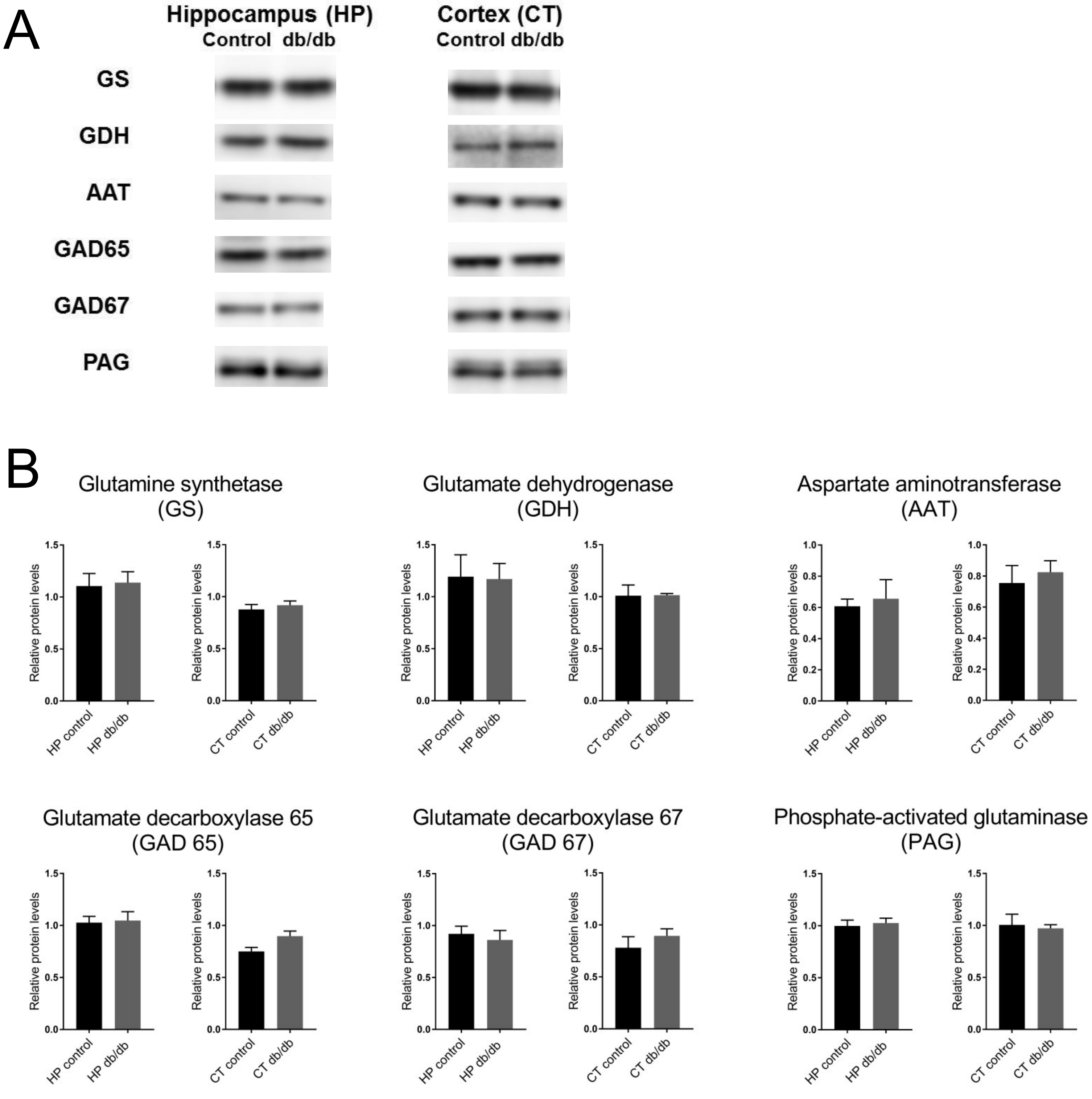

Supplementary figure 1

A: Representative Western blots of hippocampal (HP) and cerebral cortical (CT) homogenate from control and db/db mice. GS: glutamine synthetase, GDH: glutamate dehydrogenase, AAT: aspartate aminotransferase, GAD: glutamate decarboxylase, PAG: phosphate activated glutamine.

B: Semi-quantitative assessment of expression of proteins important for the glutamate-glutamine cycle. Western blots of hippocampal (HP) and cerebral cortical (CT) homogenate of control and db/db mice. Results are presented as mean ± SEM, n = 4 obtained from individual animals. Statistically significant differences were tested employing Student's t-test, $p < 0.05$.
